# Cardiac resynchronization therapy in inotrope‐dependent heart failure: a meta‐analysis

**DOI:** 10.1002/ehf2.14835

**Published:** 2024-05-06

**Authors:** Nader J. Al‐Shakarchi, Jamie S.Y. Ho, Jonathan J.H. Bray, Fabrizio D'Ascenzo, Edward Duffy, Jack Hewett, Divine Adegbie, Faizullah Khan, Niraj S. Kumar, Neal Patel, Mahmood Ahmad, Amitava Banerjee, Ikram Haq, Rui Providencia

**Affiliations:** ^1^ Mayo Clinic Rochester MN USA; ^2^ Royal Free Hospital London UK; ^3^ Oxford University Hospitals NHS Foundation Trust Oxford UK; ^4^ University of Turin Turin Italy; ^5^ University College London London UK; ^6^ East and North Hertfordshire NHS Trust Stevenage UK; ^7^ Institute for Health Informatics University College London London UK; ^8^ Barts Heart Centre, St. Bartholomew's Hospital London UK

**Keywords:** Cardiac resynchronization therapy, Heart failure, Inotrope, Meta‐analysis

## Abstract

**Aims:**

The viability of cardiac resynchronization therapy (CRT) in inotrope‐dependent heart failure (HF) has been a matter of debate.

**Methods and results:**

We searched Medline, EMBASE, Scopus, and the Cochrane Library until 31 December 2022. Studies were included if (i) HF patients required inotropic support at CRT implantation; (ii) patients were ≥18 years old; and (iii) they provided a clear definition of ‘inotrope dependence’ or ‘inability to wean’. A meta‐analysis was performed in R (Version 3.5.1). Nineteen studies comprising 386 inotrope‐dependent HF patients who received CRT (mean age 64.4 years, 76.9% male) were included. A large majority survived until discharge at 91.1% [95% confidence interval (CI): 81.2% to 97.6%], 89.3% were weaned off inotropes (95% CI: 77.6% to 97.0%), and mean discharge time post‐CRT was 7.8 days (95% CI: 3.9 to 11.7). After 1 year of follow‐up, 69.7% survived (95% CI: 58.4% to 79.8%). During follow‐up, the mean number of HF hospitalizations was reduced by 1.87 (95% CI: 1.04 to 2.70, *P* < 0.00001). Post‐CRT mean QRS duration was reduced by 29.0 ms (95% CI: −41.3 to 16.7, *P* < 0.00001), and mean left ventricular ejection fraction increased by 4.8% (95% CI: 3.1% to 6.6%, *P* < 0.00001). The mean New York Heart Association (NYHA) class post‐CRT was 2.7 (95% CI: 2.5 to 3.0), with a pronounced reduction of individuals in NYHA IV (risk ratio = 0.27, 95% CI: 0.18 to 0.41, *P* < 0.00001). On univariate analysis, there was a higher prevalence of males (85.7% vs. 40%), a history of left bundle branch block (71.4% vs. 30%), and more pronounced left ventricular end‐diastolic dilation (274.3 ± 7.2 vs. 225.9 ± 6.1 mL).

**Conclusions:**

CRT appears to be a viable option for inotrope‐dependent HF, with some of these patients seeming more likely to respond.

## Introduction

Inotrope‐dependent heart failure (HF) carries a very poor prognosis.^1^, ^2^, ^3^ To date, it is generally accepted that advanced mechanical support, including left ventricular assist devices (LVADs) or heart transplantation (HT), is the only curative means for patients with inotrope‐dependent end‐stage HF. As such, the joint 2013 American College of Cardiology and American Heart Association guidelines, as well as the European Society of Cardiology (ESC) guidelines, recommend that inotropes be used as a bridge to these therapies.^4^, ^5^ However, these are extremely invasive procedures that confer a significant risk of morbidity and mortality and are limited to a narrow pool of eligible candidates.

Cardiac resynchronization therapy (CRT) is an alternative treatment used for patients with systolic dysfunction. Several studies have shown CRT to be beneficial in the management of HF with systolic dysfunction, widened QRS complex length, and New York Heart Association (NYHA) classes II–IV.^6^ However, whether CRT is viable and safe for HF patients who require inotropic support is still a matter of debate, and its use has traditionally been limited to patients with less advanced HF due to the perceived poor prognosis of inotrope dependence and a lack of current evidence.

This perception has now started to be challenged by a meta‐analysis by Hernandez *et al*.,^7^ which demonstrated the potential benefits of CRT in these patients. These included the successful weaning of inotropes, improvements in NYHA classification, and a reduction in 1 year mortality via comparison to the REMATCH trial.^8^ However, the absence of randomized controlled trials (RCTs) and a control group were limitations.

Since the publication of the meta‐analysis by Hernandez *et al*.,^7^ further studies have been identified, and subgroup analyses comparing responders and non‐responders to CRT have now become possible, a critical factor in candidate selection. Therefore, an update is now required to assess the latest body of evidence. Therefore, we performed a meta‐analysis to evaluate the impact of CRT on clinical outcomes, echocardiographic parameters (i.e. left ventricular ejection fraction), and electrophysiologic parameters (i.e. QRS duration), and we compared the characteristics of responders and non‐responders to CRT in inotrope‐dependent HF patients.

## Methods

In accordance with the Preferred Reporting Items for Systematic Reviews and Meta‐Analyses (PRISMA) guidelines (Supporting Information, *Data S1*),^9^ we conducted a meta‐analysis of original research articles examining CRT in inotrope‐dependent HF.

### Search strategy

Medline, EMBASE, Scopus, and the Cochrane Library were searched from 1 January 1960 to 31 December 2022 with the following terms: ‘end‐stage heart failure’, ‘catecholamine‐dependent overt heart failure’, ‘inotrope‐dependent heart failure’, ‘advanced heart failure’, ‘New York Heart Association class IV’, and ‘NYHA class IV’. These terms were searched individually with ‘cardiac resynchronization therapy’ OR ‘CRT’ OR ‘biventricular device’, combined by the Boolean term ‘AND’. No language restrictions were applied. This search was repeated between 1 January 2021 and 31 December 2022 to identify more recent articles.

### Inclusion and exclusion criteria

Studies were included if they met the following inclusion criteria: (i) patients had to be dependent on inotropic support at the time of CRT implantation; (ii) if the study included other patients, outcomes had to be specifically reported on the inotrope‐dependent patients; (iii) patients included had to be more than 18 years of age; and (iv) a clear definition of ‘inotrope dependence’ or ‘inability to wean’ had to be provided. Studies with only one study arm that investigated the use of inotropes were also included. Commentaries, review articles, and studies that failed to meet the above criteria were excluded.

### Article selection

Three investigators (D. A., F. K., and N. S. K.) independently reviewed articles in three stages: by title, then abstract, and finally full review. The electronic data application, Rayyan™, was used to compile abstract information and selected articles. In instances of disagreement, consensus was reached through discussion with other co‐authors.

### Data extraction

The extracted data included study population, country, patient comorbidities, mortality, electrocardiographic and echocardiographic findings, as well as CRT and inotrope use. These data were collected through a standardized proforma. Study quality was assessed using the National Institutes of Health Quality Assessment Tool for Pre‐Post Studies with No Control Group.^10^

### Outcomes of cardiac resynchronization therapy in inotrope‐dependent heart failure patients

We assessed the following outcomes from the included studies: (i) procedural mortality; (ii) survival (at discharge, at 1 year follow‐up, and at final follow‐up); (iii) inotrope discontinuation post‐CRT; (iv) mean time to discharge post‐CRT; (v) clinical, electrocardiographic, serological, and echocardiographic parameters before and after CRT; (vi) NYHA class before and after CRT; and (vii) hospital admission for HF pre‐CRT vs. post‐CRT. A subgroup analysis of responders to CRT was also conduced; responders were defined according to the definitions provided by the included studies.

### Data analysis

Quantitative analysis was performed with R (Version 3.5.1) and SPSS (Version 26) for Kaplan–Meier analysis. Where applicable, a meta‐analysis with a random effects model was used to produce a pooled estimate of means and standard deviations or proportions. When medians and interquartile ranges (IQRs) or minimum–maximum ranges were reported, these were converted to means and standard deviations with standard formulae.^11^, ^12^ Heterogeneity between studies was quantified using the *I* ^2^ statistic (*P* ≤ 0.10 for significance), with values >50% indicating considerable heterogeneity.

## Results

### Findings from search

**Figure 1:**
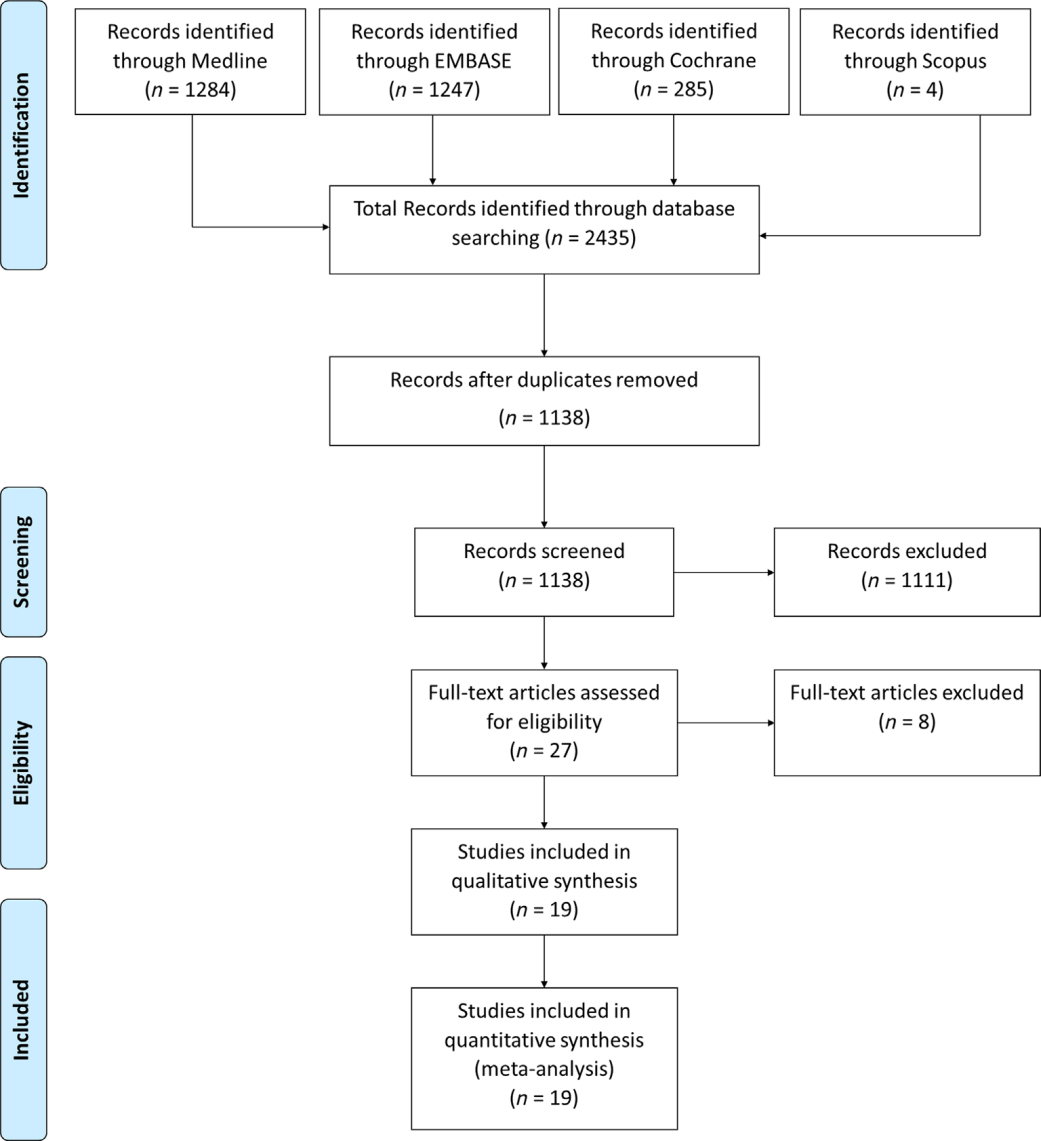
The Preferred Reporting Items for Systematic Reviews and Meta‐Analyses diagram representing the systematic literature search.

Our search identified 2435 articles. After title review, 1138 abstracts were selected, yielding 27 articles for full‐text review (*Figure* *1*). Of these, 19 met the inclusion criteria. Repeating the search between 1 January 2021 and 31 December 2022 did not identify additional relevant articles. Therefore, 19 studies were included.

### Study characteristics

Included studies were from 10 countries (*Table* *1*).^13^, ^14^, ^15^, ^16^, ^17^, ^18^, ^19^, ^20^, ^21^, ^22^, ^23^, ^24^, ^25^, ^26^, ^27^, ^28^, ^29^, ^30^, ^31^ The United States contributed the majority of studies (*n* = 7), whilst Japan contributed four studies, and the remaining countries (Canada, Spain, Israel, Korea, France, Romania, Tunisia, and Poland) all contributed one study.

**Table 1:** Overall baseline characteristics of the included studies (*n* = 19)

Variable	No. of studies	*n*	Mean (95% CI)/percentage (95% CI)
Demographics			
Age (years)	16	338	64.6 (62.2 to 67.0)
Males (%)	14	289	76.9 (70.2 to 83.1)
Follow‐up time (days)	12	257	778 (561 to 995)
Comorbidities			
Diabetes mellitus (%)	4	69	29.0 (14.0 to 46.4)
CKD (%)	3	53	41.6 (12.9 to 73.3)
Atrial fibrillation (%)	9	199	38.6 (25.7 to 52.4)
Laboratory values			
Sodium (mEq/L)	4	79	133.6 (131.9 to 135.2)
Creatinine (μmol/L)	6	117	147.8 (116.8 to 178.9)
Haemoglobin (g/dL)	3	69	11.3 (10.8 to 11.7)
BNP (pg/mL)	3	69	873 (462 to 1284)
HF aetiology			
Ischaemic cardiomyopathy (%)	13	268	58.2 (44.4 to 71.5)
Non‐ischaemic cardiomyopathy (%)	13	271	54.6 (34.3 to 74.2)
ECG and echocardiogram			
HR (b.p.m.)	3	60	81.9 (79.5 to 84.2)
SBP (mmHg)	6	110	91.2 (86.0 to 96.4)
QRS duration (ms)	14	257	168 (160 to 177)
LBBB (%)	7	105	56.0 (19.8 to 89.2)
LVEF (%)	14	278	20.2 (18.8 to 21.7)
LVEDV (mL)	6	104	224 (153 to 295)
LVESV (mL)	5	87	197 (161 to 234)
LVESD (mm)	4	110	60.7 (56.0 to 65.4)
LVEDD (mm)	6	130	69.5 (65.3 to 73.6)
CRT‐D (%)	15	234	84.5 (62.9 to 97.6)
CRT‐P (%)	15	234	15.5 (2.4 to 37.1)
IABP (%)	3	50	17.5 (7.3 to 30.1)
Hospital LOS (days)	6	99	23.4 (6.6 to 40.4)

BNP, brain natriuretic peptide; CI, confidence interval; CKD, chronic kidney disease; CRT‐D, cardiac resynchronization therapy with defibrillator; CRT‐P, cardiac resynchronization therapy with pacemaker; ECG, electrocardiogram; HF, heart failure; HR, heart rate; IABP, intra‐aortic balloon pump; LBBB, left bundle branch block; LOS, length of stay; LVEDD, left ventricular end‐diastolic diameter; LVEDV, left ventricular end‐diastolic volume; LVEF, left ventricular ejection fraction; LVESD, left ventricular end‐systolic diameter; LVESV, left ventricular end‐systolic volume; SBP, systolic blood pressure.

### Quality assessment

Study designs included a prospective cohort (*n* = 1), retrospective cohorts (*n* = 8), and case series (*n* = 10). According to the National Institutes of Health Quality Assessment Tool for Pre‐Post Studies with No Control Group, there was a low risk of bias amongst the 19 included studies (Supporting Information, *Figure* *S1*).

### Baseline characteristics

Of the included studies, a total of 386 patients with inotrope‐dependent HF were identified, and all received CRT. The mean age was 64.6 years, and the cohort was predominantly male at 76.9% (*Table* *1*). The majority of patients had HF due to ischaemic cardiomyopathy (58.2%), a left bundle branch block (LBBB) (56.0%), severely reduced left ventricular ejection fraction (20.2%), and received CRT with defibrillator (84.5%) compared with CRT with pacemaker (15.5%). More information on study baselines is presented in Supporting Information, *Table* *S1*.

### Outcomes of cardiac resynchronization therapy in inotrope‐dependent heart failure patients

**Figure 2:**
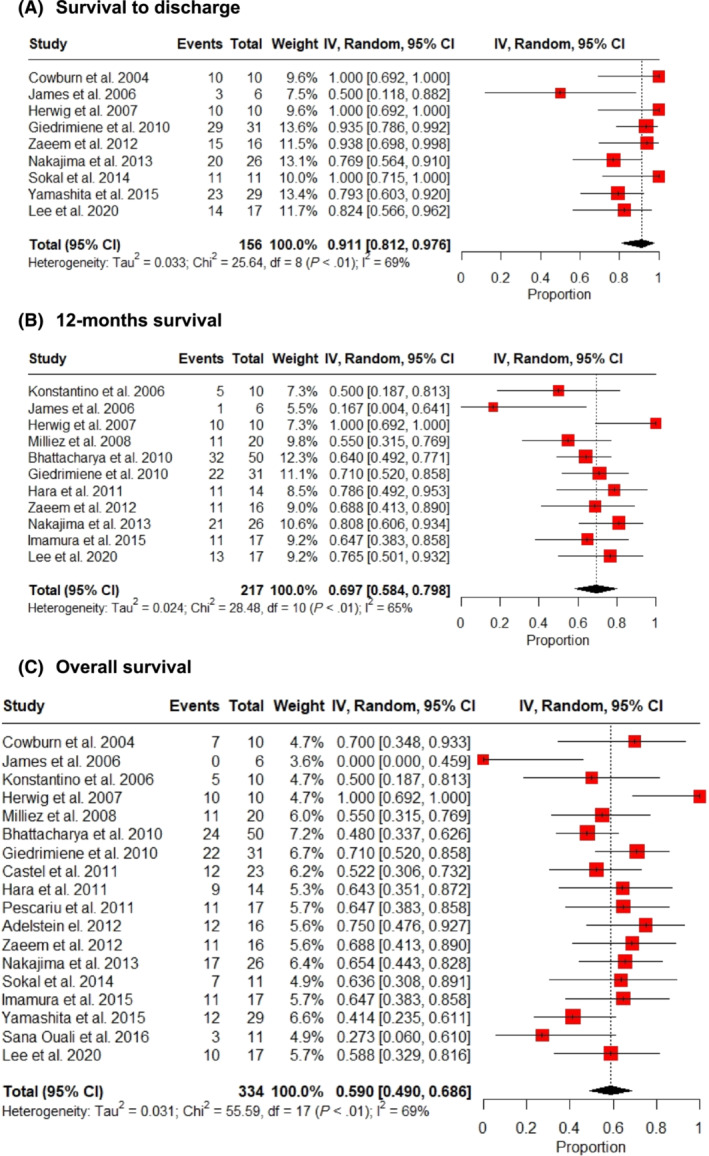
Survival outcomes post‐cardiac resynchronization therapy. (A) Discharge, (B) 12 month survival, and (C) overall survival. CI, confidence interval.

The median follow‐up was 534 days (IQR: 180 to 1500). Survival outcomes post‐CRT are shown in *Figure* *2*. The vast majority of patients survived to discharge at 91.1% [95% confidence interval (CI): 81.2% to 97.6%, *I* ^2^ = 69%; *Figure* *2* *A*],^17^, ^18^, ^20^, ^22^, ^24^, ^26^, ^29^, ^30^, ^31^ whilst 69.7% (95% CI: 58.4% to 79.8%, *I* ^2^ = 65%; *Figure* *2* *B*)^15^, ^18^, ^19^, ^20^, ^21^, ^23^, ^24^, ^25^, ^26^, ^31^ and 59.0% (95% CI: 49.0% to 68.6%, *I* ^2^ = 69%; *Figure* *2* *C*) were alive 12 months after discharge or until the end of follow‐up, respectively.^14^, ^15^, ^16^, ^17^, ^18^, ^19^, ^20^, ^21^, ^22^, ^23^, ^24^, ^25^, ^26^, ^27^, ^28^, ^29^, ^30^, ^31^ Of the nine studies that reported intra‐procedural mortality, there were no recorded deaths during CRT implantation.^15^, ^17^, ^19^, ^20^, ^25^, ^29^, ^30^

**Figure 3:**
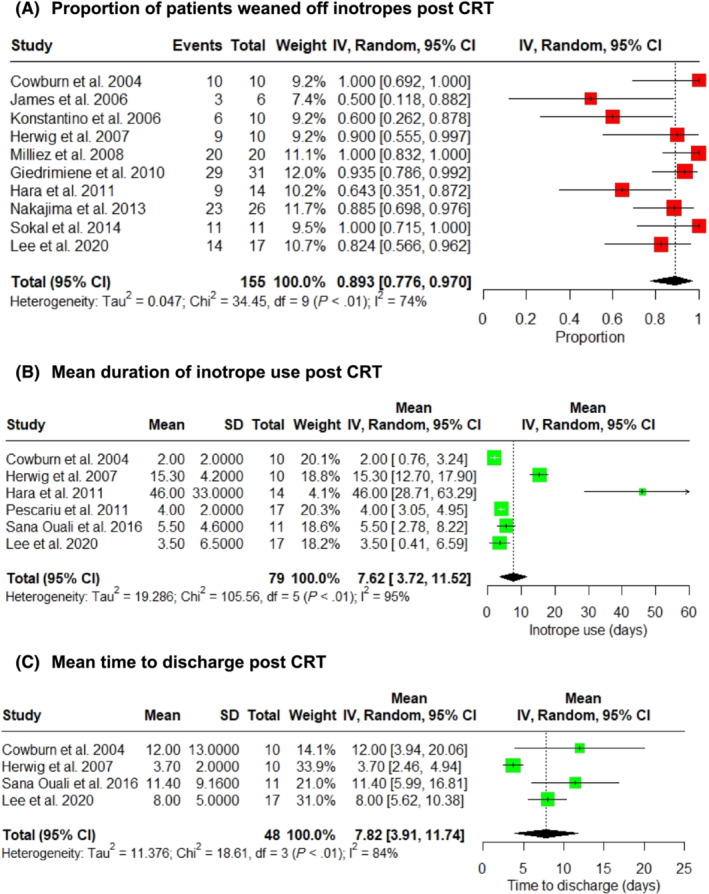
Post‐cardiac resynchronization therapy (CRT) weaning of inotropes (A), duration of inotrope use (B), and time to discharge (C) after CRT. CI, confidence interval.

Inotrope use is shown in *Figure* *3*. A significant majority of patients were weaned off inotropes post‐CRT at 89.3% (95% CI: 77.6% to 97.0%, *I* ^2^ = 74%; *Figure* *3* *A*),^17^, ^18^, ^19^, ^20^, ^22^, ^23^, ^24^, ^25^, ^26^, ^29^ with a mean duration of inotrope use of 7.6 days (95% CI: 3.7 to 11.5, *I* ^2^ = 95%; *Figure* *3* *B*),^17^, ^19^, ^20^, ^24^, ^27^, ^28^ whilst the mean time to discharge post‐CRT was 7.8 days (95% CI: 3.9 to 11.7, *I* ^2^ = 84%; *Figure* *3* *C*).^17^, ^20^, ^24^, ^28^

**Figure 4:**
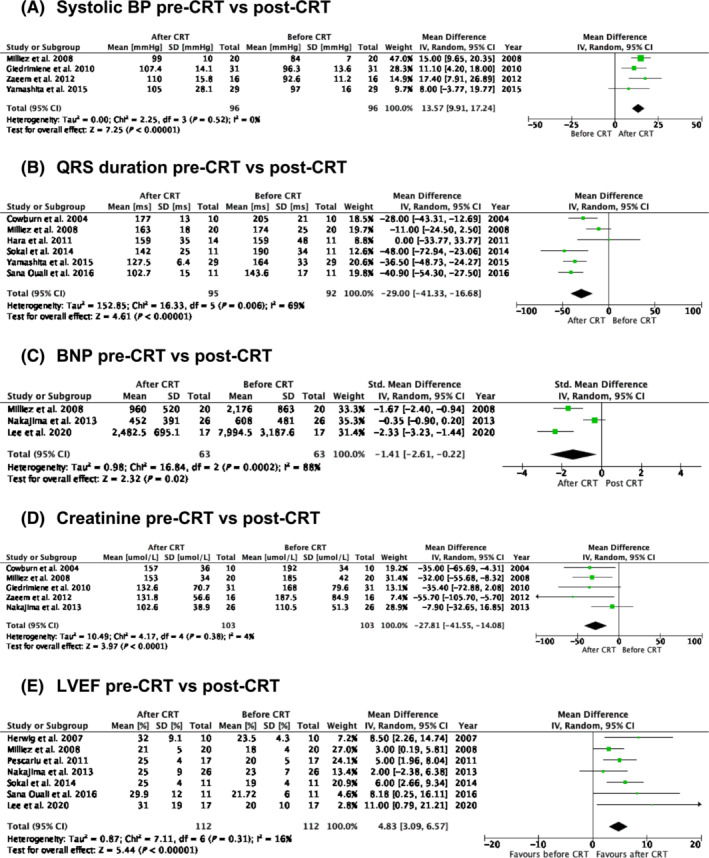
Change in clinical (A), electrocardiographic (B), serological (C, D), and echocardiographic markers (E) post‐cardiac resynchronization therapy (CRT). BNP, brain natriuretic peptide; BP, blood pressure; CI, confidence interval; LVEF, left ventricular ejection fraction.

Changes in clinical, electrocardiographic, serological, and echocardiographic markers post‐CRT are shown in *Figure* *4*. Post‐CRT mean systolic blood pressure increased by 13.5 mmHg (95% CI: 9.9 to 17.2, *I* ^2^ = 0%, *P* < 0.00001; *Figure* *4* *A*),^18^, ^25^, ^30^, ^31^ whilst mean QRS duration decreased by 29.0 ms (95% CI: −41.3 to −16.7, *I* ^2^ = 69%, *P* < 0.00001; *Figure* *4* *B*).^17^, ^19^, ^25^, ^28^, ^29^, ^30^ Serum brain natriuretic peptide (BNP) decreased by 1.4 pg/mL (95% CI: −2.61 to −0.22, *I* ^2^ = 88%, *P* = 0.02; *Figure* *4* *C*),^24^, ^25^, ^26^ mean serum creatinine decreased by 27.8 μmol/L (95% CI: −41.6 to −14.1, *I* ^2^ = 4%, *P* < 0.0001; *Figure* *4* *D*),^17^, ^18^, ^25^, ^26^, ^31^ and left ventricular ejection fraction increased 4.8% (95% CI: 3.1% to 6.6%, *I* ^2^ = 16%, *P* < 0.00001; *Figure* *4* *E*).^20^, ^24^, ^25^, ^26^, ^27^, ^28^, ^29^

**Figure 5:**
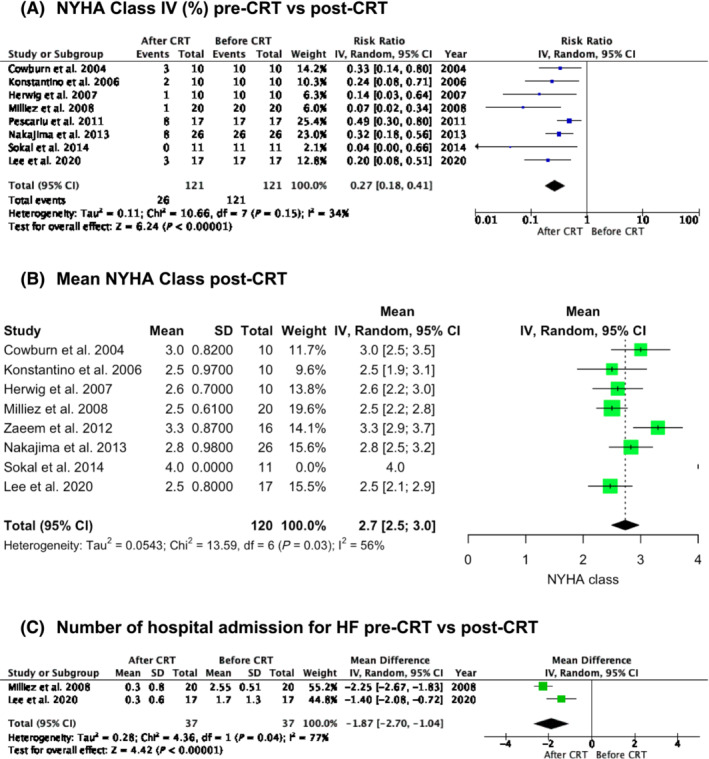
Change in heart failure (HF) severity by New York Heart Association (NYHA) class (A, B) and readmissions (C) post‐cardiac resynchronization therapy (CRT). CI, confidence interval.

HF severity and hospital readmission post‐CRT are shown in *Figure* *5*. Post‐CRT patients were significantly less likely to have NYHA class IV status (risk ratio = 0.27, 95% CI: 0.18 to 0.41, *I* ^2^ = 34%, *P* < 0.00001; *Figure* *5* *A*)^17^, ^20^, ^23^, ^24^, ^25^, ^26^, ^27^, ^29^ and had a mean NYHA class of 2.7 (95% CI: 2.5 to 3.0, *I* ^2^ = 56%; *Figure* *5* *B*).^17^, ^20^, ^23^, ^24^, ^25^, ^26^, ^29^, ^31^ The number of hospital admissions for HF post‐CRT compared with pre‐CRT decreased on average by 1.87 (95% CI: −2.70 to −1.04, *I* ^2^ = 77%, *P* < 0.00001; *Figure* *5* *C*).^24^, ^25^ Lee *et al*. compared the number of HF hospitalizations 1 year prior to CRT implantation and 1 year after,^24^ whilst Milliez *et al*. compared HF hospitalizations 15 months prior to CRT implantation with HF hospitalizations throughout the study follow‐up.^25^

### Responders vs. non‐responders to cardiac resynchronization therapy

Supporting Information, *Table* *S2* compares the characteristics of responders and non‐responders to CRT. These univariate data show a lack of significant difference in age between responders and non‐responders (65.5 ± 3.9 vs. 57.8 ± 5.8, *P* = 0.412) or the prevalence of hypertension (38.1% vs. 20.0%, *P* = 0.281), diabetes mellitus (38.1% vs. 20.0%, *P* = 0.281), non‐ischaemic cardiomyopathy (85.7% vs. 80.0%, *P* = 0.528), or atrial fibrillation (38.1% vs. 20.0%, *P* = 0.281).^19^, ^24^ However, the prevalence of LBBB was significantly greater in responders compared with non‐responders (71.4% vs. 30.0%, *P* = 0.036), and despite the lower representation, there were more women in the non‐responder group (40% vs. 16.3%, *P* = 0.015).^19^, ^24^ Left ventricular ejection fraction prior to CRT was reduced in responders compared with non‐responders (19.5% ± 0.15 vs. 24.1% ± 0.9, *P* = 0.036), whilst left ventricular end‐systolic volume (LVESV; 218.2 mL ± 1.9 vs. 175.5 mL ± 5.5, *P* = 0.016) and left ventricular end‐diastolic volume (274.3 mL ± 7.2 vs. 225.9 mL ± 6.1, *P* = 0.038) were higher in responders prior to initiation of CRT.^19^, ^24^

Responder status was defined differently across studies: Lee *et al*. defined non‐responders as patients who failed to achieve a relative reduction in LVESV of at least 15% (compared with baseline echocardiography) following CRT implantation,^24^ whilst Hara *et al*. defined non‐responders as those who could not be successfully withdrawn from intravenous inotropes in the post‐CRT implantation period.^19^

## Discussion

To the best of our knowledge, this is the largest meta‐analysis evaluating the impact of CRT on inotrope‐dependent HF patients. The most relevant findings are as follows: (i) CRT was associated with survival to discharge with successful weaning of the vast majority of patients from inotropic support; (ii) intra‐procedural mortality was extremely low; (iii) CRT prolonged survival, improved NYHA class status, reduced hospital readmissions for HF, and reduced BNP levels; and (iv) a higher prevalence of males and individuals with a history of LBBB and more pronounced left ventricular dilation, as assessed by measurement of end‐diastolic volume, was observed in the CRT responder group.

There is limited discussion of the prognosis of end‐stage HF on inotropes within the existing literature. A study by Hershberger *et al*. found that 12 month survival in inotrope‐dependent patients was only 6%.^32^ The REMATCH trial (LVAD vs. medical management), in contrast, found that 12 month survival of patients with end‐stage HF was 25%,^8^ though these patients were not necessarily inotrope dependent. These findings, therefore, appear to suggest a benefit both in symptomatic improvement and in survival when compared with optimal medical therapy.

The alternatives to CRT are HT or LVAD, either as a destination therapy or as a bridge to transplant. The 2021 ESC Guidelines now consider LVAD outcomes comparable to transplantation but acknowledge that its use is limited by adverse effects negatively affecting quality of life.^5^ These typically relate to thromboembolic or bleeding events, infection, or pump malfunction. Similarly, there are also certain challenges to HT, namely, primary graft dysfunction or complications relating to immunosuppression such as graft rejection, infection, and malignancy, not to mention the significant peri‐operative morbidity and mortality.^33^

CRT is a less invasive procedure than both HT and LVAD, with a low rate of complications in 5.6% of cases, which were most commonly the need for lead re‐intervention (2.4%), pneumothorax requiring drainage (0.9%), and infection (0.8%). Given the relative safety of CRT and the lower level of invasiveness compared with HT and LVAD, as well as its apparent survival and symptomatic benefits in patients who are dependent on inotropes, as suggested by this study, it is worth considering the possibility of utilizing CRT in patients who are ineligible for these treatments or who will be considered for this treatment after, for example, improvement of right ventricular function or of pulmonary hypertension. Furthermore, our findings that the majority of patients moved down one or two NYHA classes after receiving CRT suggest that it could be used for symptomatic relief, such as in a palliative setting.

Assessing the rates of responders vs. non‐responders, we found that patients with LBBB were more likely to respond, which is in line with the well‐known effect of CRT for correction of electrical dyssynchrony resulting from late activation of the lateral left ventricular wall.^34^ Contrary to this, however, there was no difference in mean QRS duration between responders and non‐responders. This could be due to a lack of statistical power in the sample used for the responder analysis, as numerically, CRT responders presented broader QRS complexes.

### Limitations and future directions

This meta‐analysis was limited predominantly by its relatively small sample size and significant loss of follow‐up. Perhaps most importantly, though, there is a complete lack of RCTs in this field. To compensate for that, we tried to identify case–control studies elsewhere in the literature. Unfortunately, this was limited, and the cohorts of patients in the studies we included in our review often assessed notrope‐dependent patients before and after CRT or compared inotrope‐dependent patients undergoing CRT implantation with other patients undergoing CRT but in a less advanced NYHA functional class. Only one study^30^ had a control group, which comprised age‐ and ejection fraction‐matched patients with non‐ambulatory HF who did not undergo CRT. These patients had significantly lower survival free from all‐cause death and hospitalization for HF (log rank *P* = 0.04) than those who received CRT. Cowburn *et al*.^17^ compared their cohort of inotrope‐dependent patients implanted with CRT with historical cohorts from the REMATCH study and observed that mortality at 12 months was lower in their CRT patients than in patients treated medically or with LVAD in the REMATCH cohort (30%, 75%, and 48%, respectively). However, to determine the true efficacy of CRT as rescue therapy in inotrope dependency, we would need to conduct a large RCT. As this study strongly implies that CRT carries survival and symptomatic benefits in this cohort of patients, it could be considered unethical to deprive the control group of this therapy in such a study. His bundle and left bundle branch area pacing may also play a role in this patient population as an additive or alternative treatment modality.^35^

## Conclusions

CRT appears to be a viable therapy for inotrope‐dependent HF patients. It may be an alternative to LVAD and HT in patients meeting the appropriate criteria for CRT, providing a less invasive treatment modality to a larger candidate pool with fewer inherent risks. Some patients in this population appear to respond better to CRT, namely, those with LBBB. Given the limited data available, further interventional studies, including RCTs, will be an essential next step in determining widespread implementation of CRT in these patients.

## Conflict of interest

None to declare.

## Funding

This work was funded by the British Heart Foundation (BHF) (GB) (AA/18/6/34223), the National Institute for Health and Care Research (NIHR) (GB) (NIHR129463), and the United Kingdom Research and Innovation (UKRI) and European Research Council ‐ Horizon 2020 (UKRI10103153). The corresponding author had final responsibility for the decision to submit for publication.

## Supporting information


**Data S1.** PRISMA 2020 Checklist.

**Table S1.** Study Characteristics of the included studies (n = 19).
**Table S2.** Subgroup Analysis Comparing Pre‐CRT Demographics, Comorbidities, Echocardiography and Electrocardiography Characteristics between Responders (n = 21) and Non‐Responders (n = 10) to CRT.
**Figure S1.** Risk of Bias Assessment of the included studies according to the National Institutes of Health (NIH) Quality Assessment Tool for Before‐After (Pre‐Post) Studies with No Control Group.
**Figure S2.** NYHA Classification Post‐CRT.

## References

[1] Gomes C , Terhoch CB , Ayub‐Ferreira SM , Conceição‐Souza GE , Cury Salemi VM , Chizzola PR , *et al*. Prognosis and risk stratification in patients with decompensated heart failure receiving inotropic therapy. Open Heart 2018;5:e000923. doi:10.1136/openhrt-2018-000923 30687507 PMC6330199

[2] Cuffe MS , Califf RM , Adams KF , Benza R , Bourge R , Colluci WS , *et al*. Short‐term intravenous milrinone for acute exacerbation of chronic heart failure: A randomized controlled trial. JAMA 2002;287:1541‐1547. doi:10.1001/jama.287.12.1541 11911756

[3] Abraham WT , Adams KF , Fonarow GC , Costanzo MR , Berkowitz RL , LeJemtel TH , *et al*. In‐hospital mortality in patients with acute decompensated heart failure requiring intravenous vasoactive medications: An analysis from the Acute Decompensated Heart Failure National Registry (ADHERE). J Am Coll Cardiol 2005;46:57‐64. doi:10.1016/j.jacc.2005.03.051 15992636

[4] Yancy CW , Jessup M , Bozkurt B , Butler J , Casey DE Jr , Drazner MH , *et al*. 2013 ACCF/AHA guideline for the management of heart failure: A report of the American College of Cardiology Foundation/American Heart Association Task Force on Practice Guidelines. J Am Coll Cardiol 2013;62:e147‐e239. doi:10.1016/j.jacc.2013.05.019 23747642

[5] McDonagh TA , Metra M , Adamo M , Gardner RS , Baumbach A , Böhm M , *et al*. 2021 ESC Guidelines for the diagnosis and treatment of acute and chronic heart failure: Developed by the Task Force for the diagnosis and treatment of acute and chronic heart failure of the European Society of Cardiology (ESC) with the special contribution of the Heart Failure Association (HFA) of the ESC. Eur Heart J 2021;42:3599‐3726. doi:10.1093/eurheartj/ehab368 34447992

[6] Ponikowski P , Voors AA , Anker SD , Bueno H , Cleland JGF , Coats AJS , *et al*. 2016 ESC Guidelines for the diagnosis and treatment of acute and chronic heart failure: The Task Force for the diagnosis and treatment of acute and chronic heart failure of the European Society of Cardiology (ESC)developed with the special contribution of the Heart Failure Association (HFA) of the ESC. Eur Heart J 2016;37:2129‐2200. doi:10.1093/eurheartj/ehw128 27206819

[7] Hernandez GA , Blumer V , Arcay L , Monge J , Viles‐Gonzalez JF , Lindenfeld J , *et al*. Cardiac resynchronization therapy in inotrope‐dependent heart failure patients: A systematic review and meta‐analysis. JACC Heart Fail 2018;6:734‐742. doi:10.1016/j.jchf.2018.02.016 30098968

[8] Rose EA , Gelijns AC , Moskowitz AJ , Heitjan DF , Stevenson LW , Dembitsky W , *et al*. Long‐term use of a left ventricular assist device for end‐stage heart failure. N Engl J Med 2001;345:1435‐1443. doi:10.1056/NEJMoa012175 11794191

[9] Shamseer L , Moher D , Clarke M , Ghersi D , Liberati A , Petticrew M , *et al*. Preferred Reporting Items for Systematic Review and Meta‐Analysis Protocols (PRISMA‐P) 2015: Elaboration and explanation. BMJ 2015;350:g7647. doi:10.1136/bmj.g7647 25555855

[10] National Heart, Lung, and Blood Institute . Study Quality Assessment Tools. July 2021. https://www.nhlbi.nih.gov/health‐topics/study‐quality‐assessment‐tools. Accessed 3 March 2024

[11] Hozo SP , Djulbegovic B , Hozo I . Estimating the mean and variance from the median, range, and the size of a sample. BMC Med Res Methodol 2005;5:13. doi:10.1186/1471-2288-5-13 15840177 PMC1097734

[12] Wan X , Wang W , Liu J , Tong T . Estimating the sample mean and standard deviation from the sample size, median, range and/or interquartile range. BMC Med Res Methodol 2014;14:135. doi:10.1186/1471-2288-14-13 25524443 PMC4383202

[13] Adelstein E , Schwartzman D , Gorcsan J 3rd , Saba S . Predicting hyperresponse among pacemaker‐dependent nonischemic cardiomyopathy patients upgraded to cardiac resynchronization. J Cardiovasc Electrophysiol 2011;22:905‐911. doi:10.1111/j.1540-8167.2011.02018.x 21332868

[14] Adelstein E , Bhattacharya S , Simon MA , Gorcsan J 3rd , Saba S . Comparison of outcomes for patients with nonischemic cardiomyopathy taking intravenous inotropes versus those weaned from or never taking inotropes at cardiac resynchronization therapy. Am J Cardiol 2012;110:857‐861. doi:10.1016/j.amjcard.2012.04.065 22681865

[15] Bhattacharya S , Abebe K , Simon M , Saba S , Adelstein E . Role of cardiac resynchronization in end‐stage heart failure patients requiring inotrope therapy. J Card Fail 2010;16:931‐937. doi:10.1016/j.cardfail.2010.07.253 21111981 PMC3072753

[16] Castel MA , Pérez‐Villa F , Mont L , Tolosana JM , Stiges M , Vidal B , *et al*. 118 INTERMACS level 2–3 class IV patients have worsened outcome after cardiac resynchronization therapy than stable class IV patients. J Heart Lung Transplant 2011;30:S46. doi:10.1016/j.healun.2011.01.125

[17] Cowburn P , Patel H , Jolliffe R , Wald R , Parker J . Cardiac resynchronisation therapy: An option for inotrope‐supported patients with end‐stage heart failure? Eur J Heart Fail 2005;7:215‐217. doi:10.1016/j.ejheart.2004.11.005 15701469

[18] Giedrimiene D , Zaeem F , Wencker D , Clyne C . Improvement of care and outcomes using cardiac resynchronization therapy (CRT) in patients with advanced class IV heart failure. J Interv Card Electrophysiol 2010;27:147‐266. doi:10.1007/s10840-010-9483-7 20333457

[19] Hara M , Mizuno H , Mizote I , Nakatani D , Asano Y , Sakata Y , *et al*. Clinical impact of off‐label cardiac resynchronization therapy in end‐stage heart failure patients on continuous intravenous inotrope. Clin Cardiol 2011;34:714‐720. doi:10.1002/clc.20965 22095659 PMC6652359

[20] Herweg B , Ilercil A , Cutro R , Dewhurst R , Krishnan S , Weston M , *et al*. Cardiac resynchronization therapy in patients with end‐stage inotrope‐dependent class IV heart failure. A J Cardio 2007;100:90‐93. doi:10.1016/j.amjcard.2007.02.058 17599447

[21] Imamura T , Kinugawa K , Nitta D , Hatano M , Komuro I . Should cardiac resynchronization therapy be a rescue therapy for inotrope‐dependent patients with advanced heart failure? J Card Fail 2015;21:535‐538. doi:10.1016/j.cardfail.2015.04.009 25930086

[22] James KB , Militello M , Barbara G , Wilkoff BL . Biventricular pacing for heart failure patients on inotropic support: A review of 38 consecutive cases. Tex Heart Inst J 2006;33:19‐22. PMID: 16572863 16572863 PMC1413603

[23] Konstantino Y , Iakobishvili Z , Arad O , Ben‐Gal T , Kusniec J , Mazur A , *et al*. Urgent cardiac resynchronization therapy in patients with decompensated chronic heart failure receiving inotropic therapy: A case series. Cardiology 2006;106:59‐62. doi:10.1159/000092616 16612071

[24] Lee SS , Kwon HJ , Park KM , On YK , Kim JS , Park SJ . Cardiac resynchronization therapy in New York Heart Association class‐IV patients dependent on intravenous drugs or invasive supportive treatments. ESC Heart Fail 2020;7:3109‐3118. doi:10.1002/ehf2.12940 32790157 PMC7524047

[25] Milliez P , Thomas O , Haggui A , Schurando P , Squara P , Cohen‐Solal A , *et al*. Cardiac resynchronisation as a rescue therapy in patients with catecholamine‐dependent overt heart failure: Results from a short and mid‐term study. Eur J Heart Fail 2008;10:291‐297. doi:10.1016/j.ejheart.2008.02.006 18313982

[26] Nakajima I , Noda T , Kanzaki H , Ishibashi K , Miyamoto K , Yamada Y , *et al*. Effects of cardiac resynchronization therapy in patients with inotrope‐dependent class IV end‐stage heart failure. J Arrhythm 2013;29:342‐346. doi:10.1002/joa3.12214

[27] Pescariu S , Brie D , Dumitrescu A , Sosdean R , Ionac A , Mornos C , *et al*. Is cardiac resynchronization therapy useful in patients with end‐stage heart failure patients inotrope‐supported? Eur J Heart Fail Suppl 2011;10:S122.

[28] Ouali S , Massoudi Y , Kacem S , Boughzela E . P263 Cardiac resynchronisation therapy in the treatment of end stage inotrope dependent class IV heart failure. Eur J Heart Fail 2016;18:57.

[29] Sokal A , Jędrzejczyk E , Lenarczyk R , Pluta S , Kowalski O , Pruszkowska P , *et al*. Efficacy of cardiac resynchronisation therapy in the treatment of end‐stage inotrope‐dependent heart failure patients. Kardiol Pol 2014;72:777‐782. doi:10.5603/KP.a2014.0090 24846358

[30] Yamashita S , Fukuzawa K , Yoshida A , Itoh M , Imamura K , Fujiwara R , *et al*. The effectiveness of cardiac resynchronization therapy for patients with New York Heart Association class IV non‐ambulatory heart failure. J Arrhythm 2015;31:221‐225. doi:10.1016/j.joa.2014.12.008 26336563 PMC4556084

[31] Zaeem F , Giedriemiene D , Coleman C , Crespo E , Radojevic J , Zweibel S , *et al*. CRT‐D therapy in patients with decompensated NYHA class‐four CHF. Cardiol Res Pract 2012;2012:319205. doi:10.1155/2012/319205 22900224 PMC3413993

[32] Hershberger RE , Nauman D , Walker TL , Dutton D , Burgess D . Care processes and clinical outcomes of continuous outpatient support with inotropes (COSI) in patients with refractory endstage heart failure. J Card Fail 2003;9:180‐187. doi:10.1054/jcaf.2003.24 12815567

[33] Wilhelm MJ . Long‐term outcome following heart transplantation: Current perspective. J Thorac Dis 2015;7:549‐551. doi:10.3978/j.issn.2072-1439.2015.01.46 25922738 PMC4387387

[34] Bristow MR , Saxon LA , Boehmer J , Krueger S , Kass DA , De Marco T , *et al*. Cardiac‐resynchronization therapy with or without an implantable defibrillator in advanced chronic heart failure. N Engl J Med 2004;350:2140‐2150. doi:10.1056/NEJMoa032423 15152059

[35] Hua J , Wang C , Kong Q , Zhang Y , Wang Q , Xiong Z , *et al*. Comparative effects of left bundle branch area pacing, His bundle pacing, biventricular pacing in patients requiring cardiac resynchronization therapy: A network meta‐analysis. Clin Cardiol 2022;45:214‐223. doi:10.1002/clc.23784 35128691 PMC8860481

