# Supplementary material for: Cardiac resynchronization therapy in inotrope‐dependent heart failure: a meta‐analysis
Source: ESC Heart Fail. 2024 May 6;11(5):2616–26. doi: 10.1002/ehf2.14835 (PMC11424384; doi:10.1002/ehf2.14835)
Supplement: Supplementary file 2 — Table S1. Study Characteristics of the included studies (n = 19). Table S2. Subgroup Analysis Comparing Pre‐CRT Demographics, Comorbidities, Echocardiography and Electrocardiography Characteristics between Responders (n = 21) and Non‐Responders (n = 10) to CRT. Figure S1. Risk of Bias Assessment of the included studies according to the National Institutes of Health (NIH) Quality Assessment Tool for Before‐After (Pre‐Post) Studies with No Control Group. Figure S2. NYHA Classification Post‐CRT. [file EHF2-11-2616-s002.docx]

**Supplementary Table 1: Study Characteristics of the included studies (n=19).**

| **Author, country, year** | **Study Type** | **Population** | **n** | **Age (SD, years)** | **Males (n, %)** | **Follow-Up (SD, days)** | **QRS (SD, ms)** | **LVEF (SD, %)** | **CRT-D (n, %)** | **CRT-P (n%)** | **Death (n, %)** |
| --- | --- | --- | --- | --- | --- | --- | --- | --- | --- | --- | --- |
| Cowburn et al., Canada, 2004 (17) | Case Series | Inotrope-dependent class IV HF | 10 | 71 (7) | 10 (100) | 361 (221) | 205 (21) | 20 (8) | 5 (50) | 5 (50) | 7 (70) |
| James et al., United States, 2006 (22) | Retrospective cohort | Inotrope-dependent or 30 days prior, class IV HF | 38 | 63 (13) | 29 (76) | 438 (328.5) | NR | NR | NR | NR | 0 (0) |
| Konstantino et al., Israel, 2006 (23) | Case Series | Inotrope-dependent class IV HF | 10 | 68.6 (4.8) | 9 (90) | 338 (268) | 170 (17) | NR | 8 (80) | 2 (20) | 5 (50) |
| Herwig et al., United States, 2007 (20) | Case Series | Inotrope-dependent class IV HF | 10 | 55 (13) | 9 (90) | 1088 (293) | 153 (25) | 23.5 (4.3) | 10 (100) | 0 (0) | 10 (100) |
| Milliez et al., France, 2008 (25) | Case Series | Catecholamine dependent HF | 20 | 67 (10) | 18 (90) | 540 (365) | 174 (25) | 18 (4) | 3 (15) | 17 (85) | 11 (55.0) |
| Bhattacharya et al., United States, 2010 (15) | Retrospective cohort | Inotrope-dependent class IV HF | 50 | 68.3 (5) | 36 (72) | 915 (720) | 172 (34) | 20.3 (7.4) | 50 (100) | 0 (0) | 24 (48.0) |
| Giedrimiene et al., United States, 2010 (18) | Case Series | IVD-dependent class IV HF, hospital-bound | 31 | 67.3 (10.8) | 20 (64.5) | 548 | Range 92-190 | 17.3 (5.7) | NR | NR | 22 (71.0) |
| Adelstein et al., United States, 2011 (13) | Retrospective cohort | Inotrope-dependent class IV HF due to non-ischemic cardiomyopathy, LVEF <=35%, QRS >=120 ms | 20 | NR | NR | 1399 (669) | NR | NR | 20 (100) | 0 (0) | NR |
| Castel et al., Spain, 2011 (16) | Retrospective cohort | Inotrope-dependent class IV HF | 23 | 64 (12) | NR | 423 (359) | NR | NR | NR | NR | 12 (52.2) |
| Hara et al., Japan, 2011 (19) | Retrospective cohort | Inotrope-dependent class IV HF | 14 | 58 (14) | 11 (79) | NR | 159 (48) | 20.9 (6.3) | NR | NR | 9 (64.3) |
| Pescariu et al. Romania, 2011 (27) | Case Series | Inotrope-dependent class IV HF | 17 | 63 (15) | 15 (88.2) | 608 (259) | 180 (13) | 20 (5) | 7 (7) | 10 | 11 (64.7) |
| Adelstein et al., United States, 2012 (14) | Retrospective cohort | Inotrope-dependent class IV HF due to non-ischemic cardiomyopathy, LVEF <=35%, QRS >=120 ms | 16 | 57 (9) | 11 (63) | 1430 (913) | 155 (35) | 24 (10) | 16 (100) | 0 (0) | 12 (75.0) |
| Zaeem et al., United States, 2012 (31) | Case Series | IVD-dependent advanced non-ambulatory HF | 16 | 68.1 (13.1) | 10 (63) | 548 | 164 (28) | 14.7 (5.9) | 16 (100) | 0 (0) | 11 (68.8) |
| Nakajima et al., Japan, 2013 (26) | Case Series | Inotrope-dependent class IV HF | 26 | 55 (18) | NR | 1033 (742) | 159 (38) | 23 (7) | 20 (77) | 6 (23) | 17 (65.4) |
| Sokal et al., Poland, 2014 (29) | Case Series | Inotrope-dependent class IV HF not for OHT | 11 | 63.7 (12.6) | 9 (82) | 1212 | 190 (34) | 19 (4) | 11 (100) | 0 (0) | 7 (63.6) |
| Imamura et al., Japan, 2015 (21) | Retrospective cohort | Inotrope-dependent class IV HF | 17 | NR | NR | 365 | NR | NR | 17 (100) | 0 (0) | 11 (64.7) |
| Yamashita et al., Japan, 2015 (30) | Retrospective cohort | IVD-dependent class IV HF | 29 | 67 (12) | 18 (62) | NR | 164 (33) | 23 (6) | 25 (86) | 4 (14) | 12 (41.4) |
| Sana Ouali et al. Tunisia, 2016 (28) | Case Series | inotrope-dependent HF, wide QRS complex, ineligible for urgent OHT | 11 | NR | NR | Median 534 (Range 180-1500) | 144 (17) | 21.72 (6) | 0 (0) | 11 (100) | 3 (27.3) |
| Lee et al., Korea, 2020 (24) | Prospective cohort | IVD-dependent class IV HF | 17 | 68.7 (10.9) | 11 (64.7) | 1050 (450) | 162 (36) | 20 (10) | 15 (88) | 2 (12) | 10 (58.8) |

**Supplementary Table 2: Subgroup Analysis Comparing Pre-CRT Demographics, Comorbidities, Echocardiography and Electrocardiography Characteristics between Responders (n=21) and Non-Responders (n=10) to CRT.**

Percentage of total is shown in brackets and averages are shown as mean ± standard deviation.

|  | **Responders to CRT (n=21)** | **Non-Responders to CRT (n=10)** | **p-value** |
| --- | --- | --- | --- |
| Mean Age (Years) | 65.5 ± 3.9 | 57.8 ± 5.8 | 0.412 |
| Males | 18 (85.7%) | 4 (40%) | **0.015** |
| Hypertension | 8 (38.1%) | 2 (20%) | 0.281 |
| Diabetes Mellitus | 8 (38.1%) | 2 (20%) | 0.281 |
| Non-ICM | 18 (85.7%) | 8 (80%) | 0.528 |
| AF | 8 (38.1%) | 2 (20%) | 0.281 |
| LBBB | 15 (71.4%) | 3 (30%) | **0.036** |
| LV EF (%) | 19.5% ± 0.15 | 24.1% ± 0.9 | **0.036** |
| LV ESV (mL) | 218.2 ± 1.9 | 175.5 ± 5.5 | **0.016** |
| LV EDV (mL) | 274.3 ± 7.2 | 225.9 ± 6.1 | **0.038** |
| Pre-CRT QRS (ms) | 168.7 ± 8.4 | 152.9 ± 6.1 | 0.298 |
| Post-CRT QRS (ms) | 156.7 ± 4.6 | 149.7 ± 4.4 | 0.346 |

**Abbreviations: ICM- Ischemic Cardiomyopathy, AF – Atrial Fibrillation, LBBB – Left Bundle Branch Block, LV – Left Ventricular, EF – Ejection Fraction, ESV – End-Systolic Volume, EDV – End-Diastolic Volume, CRT-D – Cardiac Resynchronization Therapy**

**
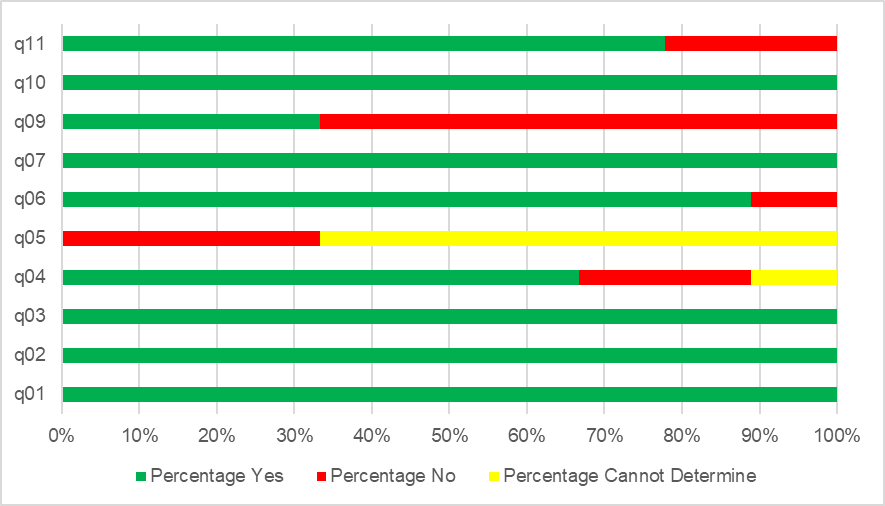
Supplementary Figure 1: Risk of Bias Assessment of the included studies according to the National Institutes of Health (NIH) Quality Assessment Tool for Before-After (Pre-Post) Studies with No Control Group.**

**Supplementary Figure 2:  NYHA Classification Post-CRT.**


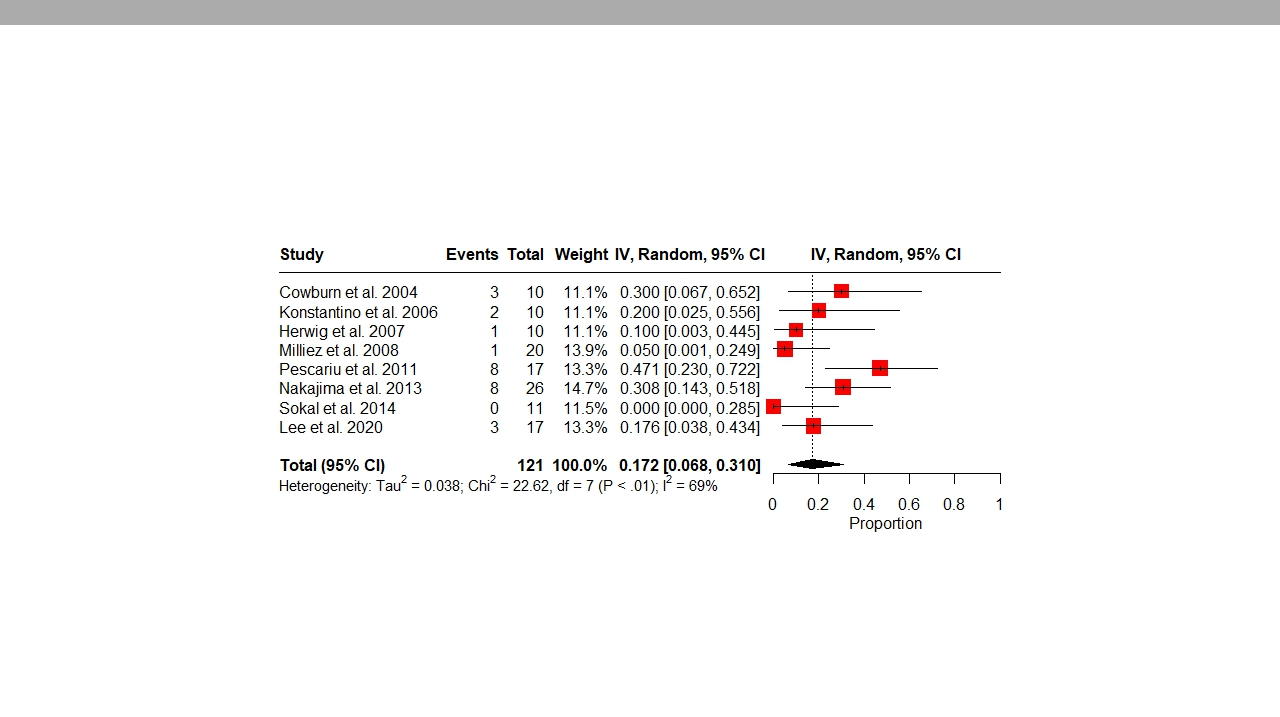


1. **Proportion NYHA IV post CRT**

1.
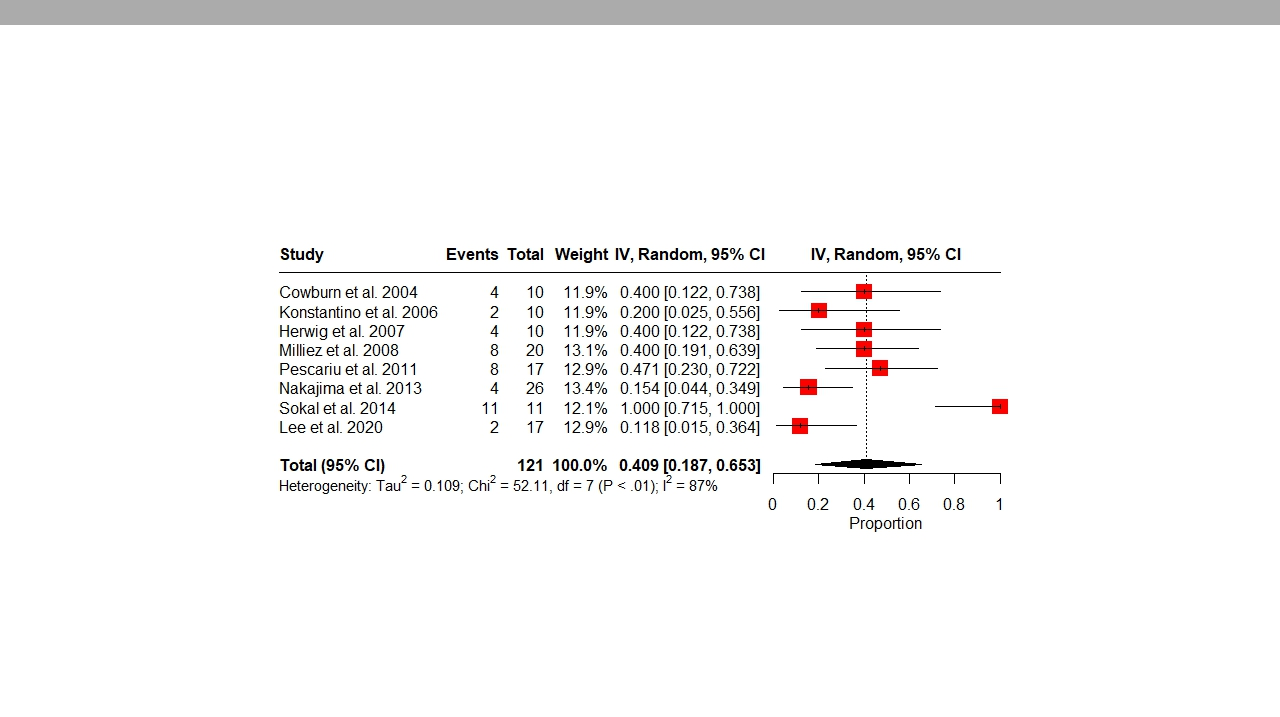
**Proportion NYHA III post CRT**

1.
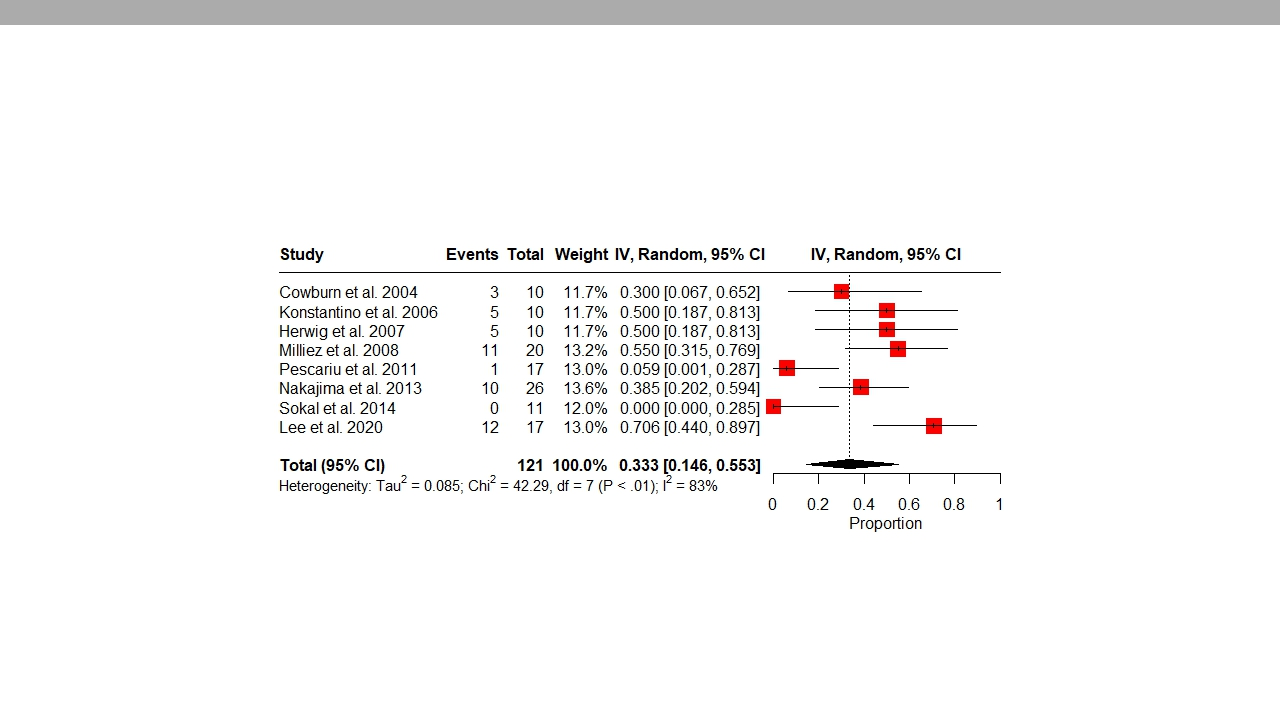
**Proportion NYHA II post CRT**

1.
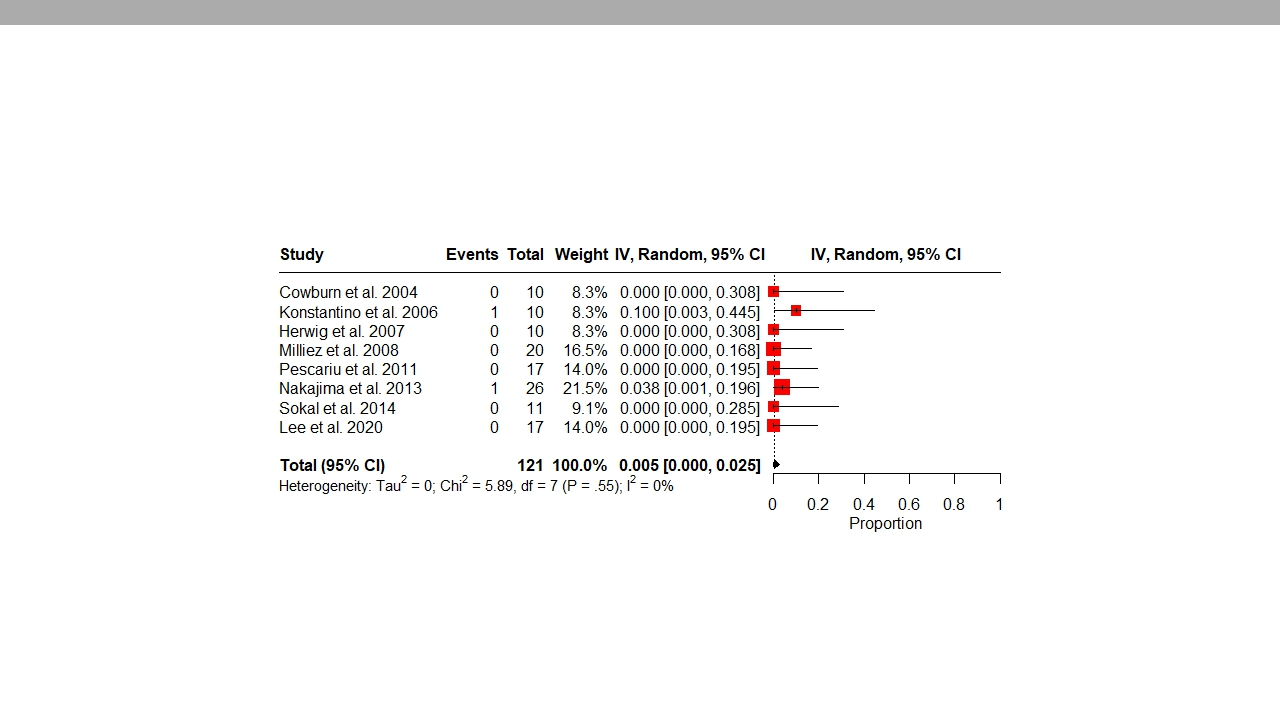
**Proportion NYHA I post CRT**
